# Removal of hypotaurine from porcine embryo culture medium does not impair development of in vitro‐fertilized or somatic cell nuclear transfer‐derived embryos at low oxygen tension

**DOI:** 10.1002/mrd.23393

**Published:** 2020-06-04

**Authors:** Paula R. Chen, Lee D. Spate, Eric C. Leffeler, Joshua A. Benne, Raissa F. Cecil, Taylor K. Hord, Randall S. Prather

**Affiliations:** ^1^ Division of Animal Sciences University of Missouri Columbia Missouri

**Keywords:** apoptosis, culture medium, hypotaurine, oxygen tension, preimplantation embryo

## Abstract

Hypotaurine (HT) is a routine component of porcine embryo culture medium, functioning as an antioxidant, but its requirement may be diminished as most embryo culture systems now use 5% O_2_ instead of atmospheric (20%) O_2_. Our objective was to determine the effects of removing HT from the culture medium on porcine preimplantation embryo development. Embryos cultured in 20% O_2_ without HT had decreased blastocyst development compared to culture with HT or in 5% O_2_ with or without HT. Notably, differences in blastocyst development or total cell number were not detected between embryos cultured in 5% O_2_ with or without HT. After culture in 5% O_2_ without HT and embryo transfer, healthy fetuses were retrieved from two pregnancies on Day 42, confirming in vivo developmental competence. Transcript abundance of proapoptotic markers was decreased in embryos cultured without HT regardless of oxygen tension; however, assays for apoptosis did not demonstrate differences between groups. Additionally, no differences were observed in the development or apoptosis of somatic cell nuclear transfer‐derived embryos cultured in 5% O_2_ with or without HT. With decreased utility in 5% O_2_, removing HT from porcine embryo culture medium would also have economic advantages because it is undoubtedly the most expensive component.

## Graphical abstract

Hypotaurine is an antioxidant added to porcine embryo culture medium, and it is the most expensive component of the formulation. However, removal of hypotaurine did not decrease development of in vitro‐fertilized or somatic cell nuclear transfer‐derived embryos to the blastocyst stage when culture was conducted in 5% O_2_, and pregnancies were established after transferring embryos cultured without hypotaurine. Transcript abundance of proapoptotic markers was decreased in embryos cultured without hypotaurine, but differences in apoptosis between embryos cultured with or without hypotaurine were not observed at the cellular level.

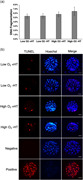

## 1 INTRODUCTION

Hypotaurine (HT) is an aminosulfinic acid that is added to embryo culture media to act as a reactive oxygen species (ROS) scavenger to prevent cellular damage and improve development. Moreover, addition of HT to embryo culture media was another step toward mimicking in vivo antioxidant systems. Secretion of HT into follicular and oviductal fluids has been detected in several mammalian species, including pigs (Guérin & Ménézo, 1995). Within the microenvironment, HT is oxidized to taurine to quench hydroxyl radicals (^•^OH) which are more commonly generated by in vitro systems than in the reproductive tract due to manipulation in atmospheric oxygen tension (20%), direct exposure to light and radiation, and contamination of culture media with metal ions, most commonly Fe^+2^ (Guérin, El Mouatassim, & Ménézo, 2001). In hamster embryos, HT was required for development to proceed to at least the eight‐cell stage, but development to the morula and blastocyst stages was not dependent upon the concentration (0.1, 1, or 10 mM) of HT (Barnett & Bavister, 1992). Addition of 5 mM HT to NSCU‐23 significantly improved development to the blastocyst stage in porcine embryos (Petters & Reed, 1991), and bovine blastocyst development increased after culture with 0.5‐ to 2‐mM HT on monolayers of Vero cells (Guyader‐Joly et al., 1998).

The majority of studies demonstrating the beneficial effects of HT during embryo culture were conducted in 5% CO_2_ in air (20% O_2_). However, preimplantation embryo development in vivo occurs at 1.5–8.5% O_2_ depending on the species, and oxygen tension decreases around the period of blastocyst development and implantation (Fischer & Bavister, 1993; Ufer & Wang, 2011). Culture in 5% O_2_ compared to 20% O_2_ has been shown to improve blastocyst development and total cell numbers in embryos of many different species (Batt, Gardner, & Cameron, 1991; Booth, Holm, & Callesen, 2005; Karagenc, Sertkaya, Ciray, Ulug, & Bahçeci, 2004; Lim, Reggio, Godke, & Hansel, 1999; Redel et al., 2011) as well as live birth rates after in vitro fertilization (IVF) in humans (Meintjes et al., 2009). Furthermore, a meta‐analysis of 21 studies confirmed a modest increase in the number of clinical pregnancies after transferring human embryos that were cultured in 5% O_2_; however, more trials are required to confirm the results (Nastri et al., 2016). Redel et al. (2011) observed that the transcriptional profiles of porcine embryos cultured in 5% O_2_ were characteristic of the Warburg effect (WE) with an increased message for enzymes of glycolysis. The WE describes the shuttling of glycolytic intermediates away from the tricarboxylic acid cycle and towards the pentose phosphate pathway and lactic acid formation in rapidly proliferating cells to support macromolecule synthesis (Warburg, 1956). This metabolic explanation of reduced respiration is in agreement with the fact that porcine embryos cultured in 5% O_2_ have decreased ROS accumulation and DNA damage compared to embryos cultured in 20% O_2_ (Kitagawa, Suzuki, Yoneda, & Watanabe, 2004).

Therefore, when culturing porcine embryos in 5% O_2_, we hypothesized that addition of HT to the embryo culture medium was unnecessary. First, we investigated the effects of culturing embryos with or without HT in 5% or 20% O_2_ on embryo cleavage and blastocyst development. Embryo transfers were performed by using blastocysts that developed after culturing without HT in 5% O_2_ to confirm that this treatment could establish pregnancies. Subsequently, the abundance of transcripts related to HT synthesis, oxidative stress, and apoptosis, as well as incidences of apoptosis, were determined in the blastocysts. Finally, developmental parameters were reassessed in somatic cell nuclear transfer (SCNT)‐derived embryos cultured with or without HT in 5% O_2_ as these embryos are more prone to stress and damage during micromanipulation procedures.

## 2 RESULTS

### 2.1 Development is not impaired by HT removal at low oxygen tension

Culture with (+) or without (−) HT was investigated to determine the impact on porcine embryo development at 5% (low) O_2_ and 20% (high) O_2_. Between all four groups, no differences in cleavage on D2 were detected (Table 1). Embryos cultured in low O_2_ −HT or +HT demonstrated no differences in development to the blastocyst stage on D6 (41.1 ± 2.8% vs. 42.4 ± 2.0%) or the total number of nuclei in the blastocyst‐stage embryos (50.5 ± 1.5 vs. 50.0 ± 1.8). However, embryos cultured in high O_2_ −HT had significantly decreased blastocyst development compared to all other groups, and embryos cultured in high O_2_ +HT had a decreased number of nuclei compared to those cultured in low O_2_ −HT (Table 1). Therefore, HT is potentially dispensable in low O_2_, but it is beneficial for preimplantation development in high O_2_.

**Table 1:** Development of IVF embryos cultured in different oxygen tensions and with or without HT

Treatment^a^	Percentage cleavage (D2) ± SEM	Percentage blastocyst (D6) ± SEM	Total number of nuclei ± SEM
Low O_2_ +HT	63.8 ± 2.8	42.4 ± 2.0^a^	50.0 ± 1.8^a,b^
Low O_2_ −HT	61.8 ± 5.3	41.1 ± 2.8^a^	50.5 ± 1.5^a^
High O_2_ +HT	56.7 ± 2.2	41.0 ± 3.1^a^	45.5 ± 1.2^b^
High O_2_ −HT	57.5 ± 4.7	31.7 ± 2.9^b^	48.0 ± 1.2^a,b^

*Note*: Values with different superscripts (a,b) indicate statistical differences (*p* < .05).

Abbreviations: HT, hypotaurine; IVF, in vitro fertilization; SEM, standard error of the mean.

^a^ *n* = 650 presumptive zygotes across seven replicates.

### 2.2 Establishment of pregnancy after transferring embryos cultured without HT

**Figure 1:**
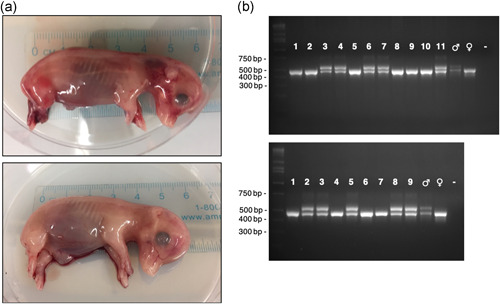
D42 fetal collections after transfer of embryos cultured in low O_2_ without hypotaurine. (a) Fetuses from Pregnancy 1 (top) and Pregnancy 2 (bottom). (b) Sex determination assay to confirm observed sexes by external genitalia. Amplicon size for X is 485 base pairs (bp) and for Y is 545 bp

To ensure that porcine embryos cultured in low O_2_ −HT could establish pregnancies, two embryo transfers were performed with 60 IVF‐derived D5 blastocysts and morulae each. Fetuses were collected on D42 of gestation. The pregnancies yielded 11 and 9 fetuses, respectively, and the sexes and average crown–rump lengths were measured (Table 2). Importantly, all fetuses appeared to be healthy with no signs of absorption (Figure 1a). Sexes determined by external genitalia were confirmed by using a polymerase chain reaction (PCR)‐based sex determination assay (Figure 1b). These data confirm that culturing embryos without HT can result in the successful establishment of pregnancy.

**Table 2:** Fetal collections on D42 after transferring embryos cultured in 5% O_2_ without HT

Pregnancy	Number of fetuses	Average crown–rump length (cm) ± SEM	Males	Females
1	11	5.9 ± 0.13	5	6
2	9	6.1 ± 0.08	5	4

Abbreviations: HT, hypotaurine; SEM, standard error of the mean.

### 2.3 Effect of HT removal on transcript abundance, caspase activity, and DNA fragmentation

**Figure 2:**
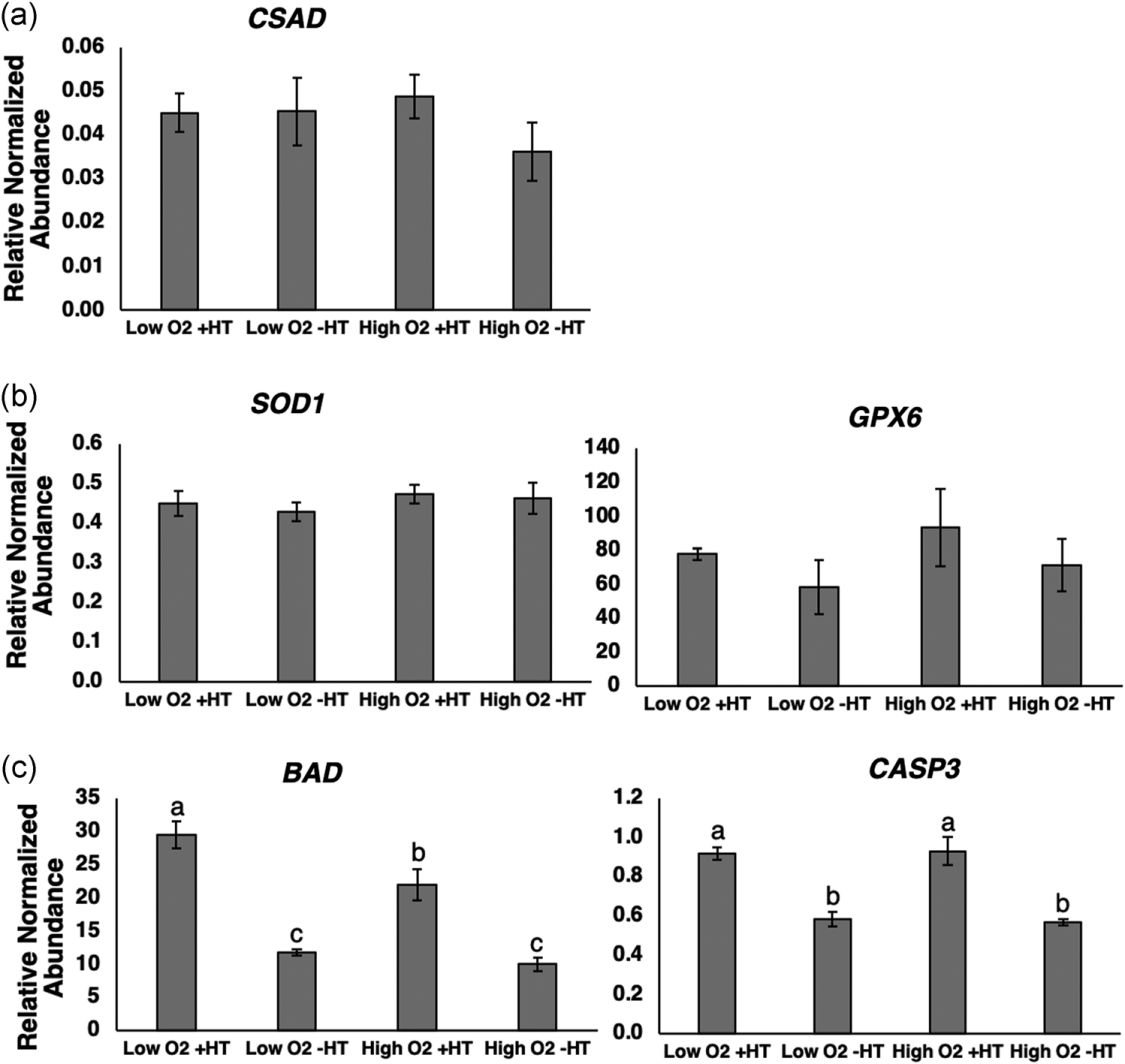
Relative abundance of transcripts involved in (a) hypotaurine (HT) synthesis, (b) oxidative stress, and (c) apoptosis from embryos cultured in low O_2_ +HT, low O_2_ −HT, high O_2_ +HT, or high O_2_ −HT determined by quantitative polymerase chain reaction. Data are presented as mean ± standard error. Different lowercase letters indicate statistical differences (*p* < .05)

Abundance of transcripts related to HT synthesis (cysteine sulfinic acid decarboxylase [*CSAD*]), oxidative stress (superoxide dismutase 1 [*SOD1*], glutathione peroxidase 6 [*GPX6*]), and apoptosis (BCL‐2 associated agonist of cell death [*BAD*], caspase 3 [*CASP3*]) were measured by quantitative PCR (qPCR). No differences in transcript abundance were detected for *CSAD*, *SOD1*, or *GPX6* between any of the groups (Figure 2a,b). Embryos cultured in low O_2_ or high O_2_ −HT had significantly decreased abundance of *BAD* and *CASP3* compared to embryos cultured in low O_2_ or high O_2_ +HT (Figure 2c). Furthermore, embryos cultured in low O_2_ +HT had a significantly increased abundance of *BAD* compared to embryos cultured in high O_2_ +HT. Thus, the transcript abundances of selected proapoptotic markers decreased after HT removal regardless of oxygen tension, indicating that HT may promote apoptosis.

**Figure 3:**
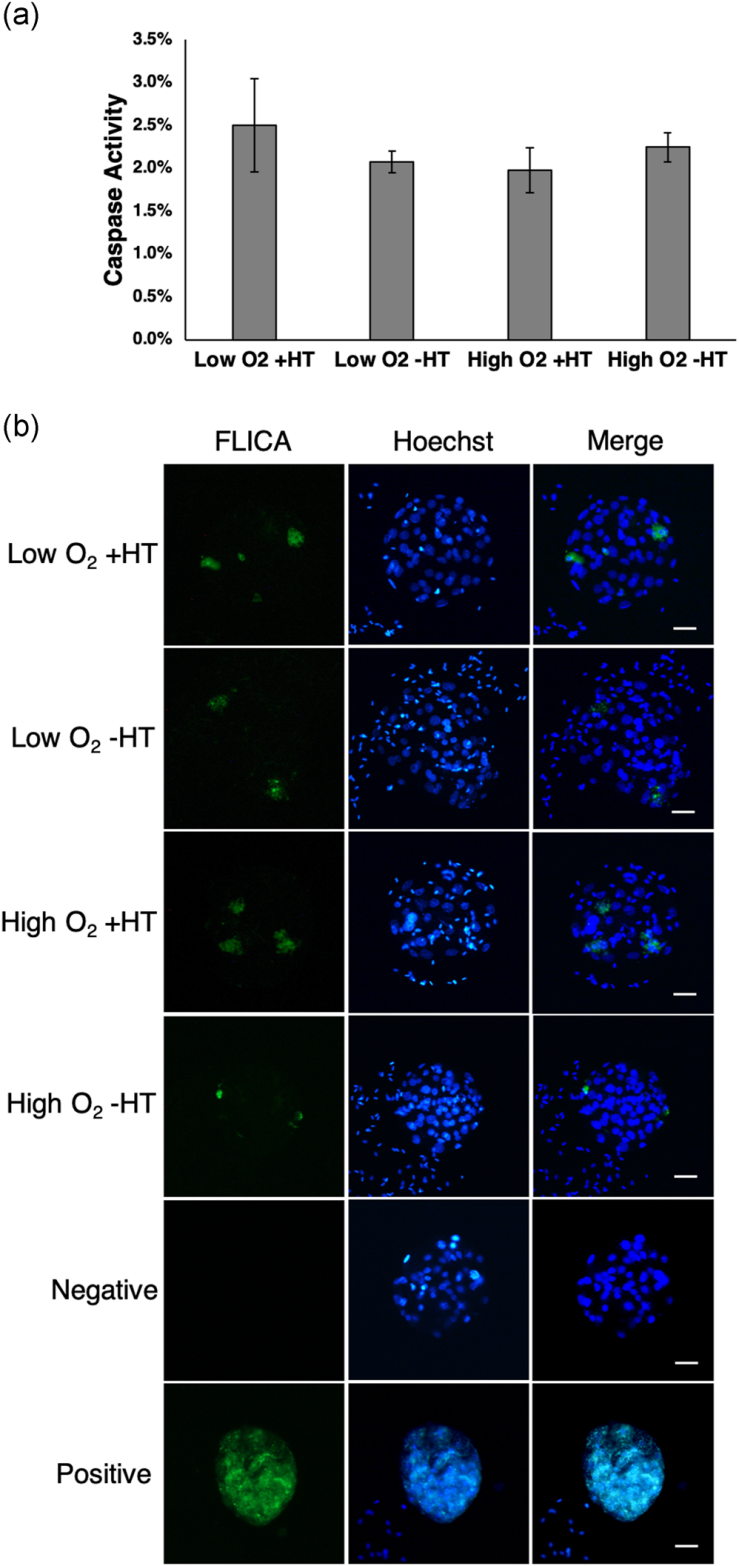
Effects of oxygen tension and presence of hypotaurine on caspase‐3 and caspase‐7 activation. (a) Caspase activation determined by staining with fluorochrome inhibitor of caspases (FLICA). Values were determined from four replicates (*n* = 40–50 embryos per treatment). Data are presented as mean ± standard error. (b) Representative images of FLICA‐positive cells in D6 blastocyst‐stage embryos from each treatment. Nuclei are stained with Hoechst 33342. Scale bars = 50 μm

**Figure 4:**
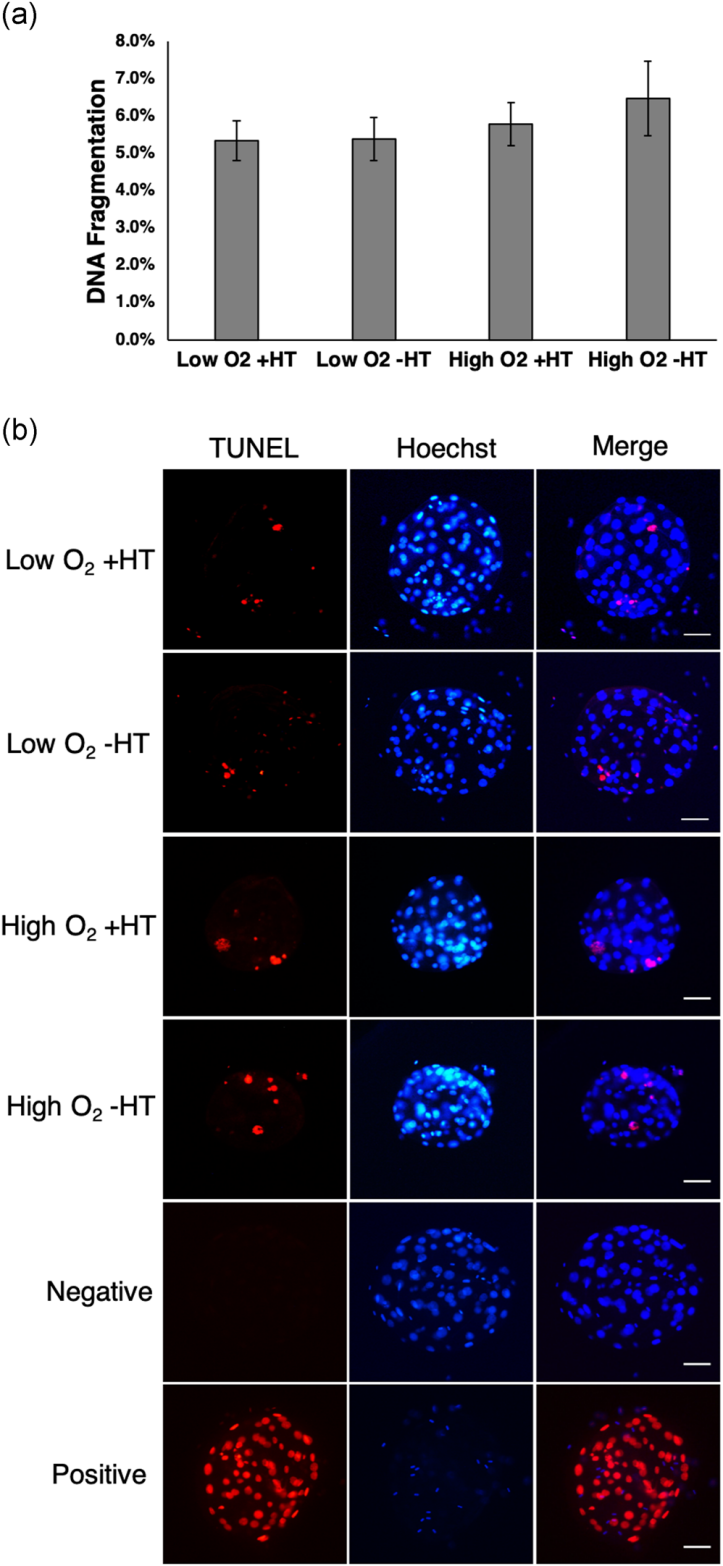
Effects of oxygen tension and presence of hypotaurine on DNA fragmentation. (a) DNA damage determined by staining with terminal deoxynucleotidyl transferase dUTP nick‐end labeling (TUNEL). Values were determined from four replicates (*n* = 40–50 embryos per treatment). Data are presented as mean ± standard error. (b) Representative images of TUNEL‐positive nuclei in D6 blastocyst‐stage embryos from each treatment. Nuclei are stained with Hoechst 33342. Scale bars = 50 μm

Two assays for apoptosis were performed to determine if removal of HT decreased incidence of this process. No differences in the activity of caspases 3 and 7 were detected between any groups after fluorochrome inhibitor of caspases (FLICA) staining (Figure 3a,b). Embryos cultured in low O_2_ +HT or −HT had caspase activity indexes of 2.5 ± 0.5% and 2.1 ± 0.1%, respectively. Embryos cultured in high O_2_ +HT or −HT had caspase activity indexes of 2.0 ± 0.3% and 2.3 ± 0.2%, respectively. Analysis of DNA damage by terminal deoxynucleotidyl transferase dUTP nick‐end labeling (TUNEL) staining revealed no difference between groups (Figure 4a,b). Embryos cultured in low O_2_ +HT or −HT had DNA fragmentation indexes of 5.4 ± 0.5% and 5.4 ± 0.6%, respectively. Embryos cultured in high O_2_ +HT or −HT had apoptotic indexes of 5.8 ± 0.6% and 6.5 ± 1.0%, respectively.

### 2.4 SCNT‐derived embryos exhibit normal development without HT in the medium

In addition to IVF‐produced embryos, SCNT‐derived embryos were cultured in low O_2_ +HT or −HT to assess cleavage and blastocyst development as well as DNA fragmentation. No differences in cleavage on D2 were detected (Table 3). Development to the blastocyst stage on D6 was not different for embryos cultured +HT (37.1 ± 8.4%) or −HT (43.3 ± 6.4%). Interestingly, the total number of nuclei was significantly decreased in embryos cultured +HT (47.7 ± 0.6) compared to those cultured −HT (51.4 ± 0.8). Moreover, the percentage of DNA fragmentation determined by TUNEL staining was not different between the two groups. Removal of HT from the culture medium did not appear to be detrimental to the development of SCNT‐derived embryos.

**Table 3:** Development of SCNT embryos cultured in 5% O_2_ with or without HT

Treatment	Percentage cleavage (D2) ± SEM	Percentage blastocyst (D6) ± SEM	Total number of nuclei ± SEM	Percentage TUNEL‐positive nuclei ± SEM
Low O_2_ +HT	84.4 ± 2.9	37.1 ± 8.4	47.7 ± 0.6^b^	5.5 ± 1.7
Low O_2_ −HT	81.9 ± 4.0	43.3 ± 6.4	51.4 ± 0.8^a^	5.8 ± 0.6

*Note*: *n* = 187 reconstructed embryos across four replicates.

Abbreviations: HT, hypotaurine; SCNT, somatic cell nuclear transfer; SEM, standard error of the mean; TUNEL, terminal deoxynucleotidyl transferase dUTP nick‐end labeling.

Values with different superscripts (a,b) indicate statistical differences (*p* < .05).

## 3 DISCUSSION

Embryos produced in vitro are subjected to different sources of free radicals which can result in the accumulation of damage and developmental arrest or apoptosis. Of the free radicals, reactive oxygen species (ROS) are the most common with endogenous sources, including oxidative phosphorylation and NADPH oxidases, and exogenous sources, including oxygen tension, light, metal ions, and ionizing radiation. Thus, embryo culture media have been formulated to contain various antioxidants to counteract the rise in ROS (Guérin et al., 2001). Specifically, HT is a well‐known scavenger of hydroxyl radicals (^•^OH) that reduces lipid peroxidation in spermatozoa (Alvarez & Storey, 1983; Aruoma, Halliwell, Hoey, & Butler, 1988). Petters and Reed (1991) reported that addition of HT to porcine embryo culture medium improved the development of one‐ and two‐cell stage embryos to the blastocyst stage. Thereafter, HT has been routinely added to porcine embryo culture medium as a protective measure against ROS. However, with the advent of embryo culture being performed in 5% O_2_, the necessity for antioxidants, including HT, in the medium decreases because of reduced exposure to ROS. Furthermore, HT is the most expensive component of porcine embryo culture medium, accounting for 68% of the cost of making MU2 on a per‐mL basis (Spate, Brown, Redel, Whitworth, & Prather, 2015). Therefore, its removal from formulations would have an economic advantage for embryo culture laboratories as well.

The current study investigated the effects of culturing porcine embryos in 5% (low) or 20% (high) O_2_ and in the presence (+) or absence (−) of HT. Development to the blastocyst stage on Day 6 was not different between embryos cultured in low O_2_ +HT, low O_2_ −HT, and high O_2_ +HT but was decreased when embryos were cultured in high O_2_ −HT. Thus, exposure to atmospheric oxygen tension without the protective effects of antioxidants is deleterious for preimplantation development. Total cell numbers were decreased when embryos were cultured in high O_2_ +HT compared to all other treatments which could be the result of more apoptotic cells in these blastocysts, and no differences in total cell numbers were detected between groups cultured in low O_2_. Suzuki, Yoshioka, Sakatani, and Takahashi (2007) observed that porcine embryos cultured in 5% O_2_ with or without HT did not demonstrate differences in blastocyst development or total cell numbers. Therefore, HT did not have a detectable impact on embryo development at 5% O_2_.

Moreover, developmental competence in vivo was assessed by performing two embryo transfers after culture to Day 5 in low O_2_ −HT. Due to the fact that “good quality” blastocysts in culture do not always establish pregnancies (Redel et al., 2016), embryo transfers were an important component of this study. Both pregnancies were terminated on Day 42 due to space considerations in the facility. One gilt had 11 fetuses and the other contained 9 fetuses, none of which were being absorbed, indicating the successful establishment of pregnancy by using embryos cultured in the absence of HT. Although control transfers by using embryos cultured with HT were not included in this study, previous work in our laboratory has demonstrated that these embryos establish pregnancies as well (Yuan et al., 2017). Direct comparisons with Yuan et al. (2017) cannot be made, but the average litter size of embryos cultured with HT was 8.6 piglets after 15 transfers with a 46.7% pregnancy rate. Additionally, male embryos have been shown to develop faster in culture and contain more cells in the blastocyst‐stage embryos compared to females which can impact the sex ratio after the transfer (Cassar, de la Fuente, Yu, King, & King, 1995). Despite the fact that the current study did not include sufficient pregnancies to determine alterations in sex ratio, the observed ratio of male to female fetuses from both pregnancies was approximately 1:1.

The abundance of transcripts involved in HT synthesis, oxidative stress, and apoptosis was measured to determine if removing HT from the culture medium affected these processes. Interestingly, the abundance of proapoptotic transcripts, BCL‐2 associated agonist of cell death (*BAD*) and caspase 3 (*CASP3*) was increased in embryos cultured with HT regardless of oxygen tension. Additionally, expression of *BAD* was significantly increased in embryos cultured in low O_2_ +HT compared to low O_2_ −HT, which potentially indicated that HT stimulates apoptosis. BAD promotes apoptosis by binding antiapoptotic regulators, Bcl‐xL and Bcl‐2, to allow for the opening of the mitochondrial permeability transition pore and release of cytochrome *c* (Howells, Baumann, Samuels, & Finkielstein, 2011). CASP3 is an executioner caspase (cysteine‐aspartic acid protease) that is activated by the apoptosome, comprised of apoptosis‐activating factor 1 and caspase‐9, for activating other caspases and proteolytically degrade the cell (McIlwain, Berger, & Mak, 2013). The product of HT oxidation, taurine, has been shown to decrease the formation of the apoptosome, thereby inhibiting the apoptosis cascade (Takatani et al., 2004). Moreover, HT addition to cryopreservation medium for human sperm reduced the percentage of apoptotic sperm after thawing (Brugnon et al., 2013). Apoptosis assays were utilized in the current study to assess the effects of removing HT instead of ROS assays because of inconsistencies in the latter. For example, the most common ROS probe, dichloro‐dihydro‐fluorescein diacetate (DCFH‐DA), forms a free radical during its oxidation that can generate more superoxide ions, and cytochrome *c* released during apoptosis can oxidize the probe to falsely increase the fluorescence signal (Kalyanaraman et al., 2012). Apoptosis can be detected in single cells by using the FLICA assay which uses a caspase‐3 inhibitor linked to a carboxyfluorescein that covalently binds to active caspase‐3 and caspase‐7 heterodimers. Moreover, TUNEL staining adds fluorochrome‐label dUTP to the 3′ ends of fragmented DNA to observe nuclei with DNA damage as another measure of apoptosis. Assessing apoptosis by FLICA and TUNEL staining in the blastocysts revealed no differences between the four treatment groups; therefore, we could not detect a decrease in the incidence of apoptosis after removal of HT from the culture medium. However, Suzuki et al. (2007) used a comet assay and noted that addition of 5‐mM HT to porcine embryo culture medium decreased DNA fragmentation in Day 3 cleaved embryos cultured in 5% O_2_, which could potentially reflect stage‐specific requirements of medium components or source of oocytes.

Embryos generated by somatic cell nuclear transfer (SCNT) are more susceptible to damage during micromanipulation procedures and exhibit developmental delays (Martin et al., 2007). Furthermore, activation by chemical methods has been shown to increase apoptotic indexes in Day 7 SCNT‐derived porcine blastocysts (Im et al., 2006). The effect of removing HT from the culture medium was assessed in SCNT‐derived porcine embryos after culture in low O_2_, and no differences were observed in percentages of embryos cleaved on Day 2 and development to the blastocyst stage on Day 6. Additionally, the percentage of nuclei with DNA fragmentation detected by TUNEL staining was not different, confirming that removing HT from the medium does not negatively impact SCNT‐derived embryos.

Although HT supplementation to embryo culture medium has previously been demonstrated to be beneficial for several species, it may not be necessary in modern culture systems. Removal of HT from our porcine embryo culture medium did not appear to decrease development in low O_2_ but was beneficial in high O_2_. In addition, healthy fetuses were obtained after transferring embryos cultured in low O_2_ −HT, affirming in vivo developmental competence. The abundance of proapoptotic transcripts was decreased in embryos cultured without HT regardless of oxygen tension; however, apoptosis assays did not reveal differences. This is in agreement with the fact that messenger RNA (mRNA) and protein levels are not always correlated and rely on several factors, such as rates of degradation and activity or availability of translational machinery (Y. Liu, Beyer, & Aebersold, 2016). Moreover, caspases must dimerize and be processed before activation as a measure of preventing unintentional apoptosis (H. Liu, Chang, & Yang, 2005). SCNT‐derived embryos developed normally when cultured in low O_2_ –HT, suggesting that HT can be removed from porcine zygote medium (PZM) variants. As the removal of HT from porcine embryo culture medium is economically advantageous, its effects should be reassessed in other systems, such as the NCSU series of culture media, to determine widespread applicability.

## 4 MATERIALS AND METHODS

### 4.1 Chemical components

All chemicals were purchased from Sigma Chemical Company (St. Louis, MO) unless stated otherwise.

### 4.2 Ethics statement

The use of live animals and collection of ovaries from prepubertal gilts were in accordance with the approved protocol and standard operating procedures by the Animal Care and Use Committee of the University of Missouri.

### 4.3 Oocyte collection and maturation

Ovaries were collected from prepubertal gilts at a local abattoir (Smithfield Foods, Inc., Milan, MO). Follicles with diameters of 3–6 mm were aspirated by using an 18‐gauge needle attached to a 10‐ml disposable syringe. Cumulus‐oocyte complexes with a uniform cytoplasm and at least three layers of cumulus cells were collected and placed in maturation medium (TCM‐199 medium supplemented with 0.1% polyvinyl alcohol [PVA], 3.05 mM d‐glucose, 0.91 mM sodium pyruvate, 10 µg/ml of gentamicin, 0.57 mM cysteine, 10 ng/ml of epidermal growth factor, 0.5 µg/ml follicle‐stimulating hormone, and 0.5 µg/ml luteinizing hormone) supplemented with FLI (40 ng/ml FGF2, 20 ng/ml LIF, and 20 ng/ml IGF1) and matured for 42–44 hr at 38.5°C in a humidified incubator with an atmosphere of 5% CO_2_ in air (Yuan et al., 2017). Cumulus cells were removed from matured oocytes by vortexing for 2 min in 0.1% (w/v) hyaluronidase in Tyrode's lactate 4‐(2‐hydroxyethyl)‐1‐piperazineethanesulfonic acid (TL‐HEPES)‐buffered saline with 0.1% PVA. Denuded oocytes were placed in manipulation medium (9.50 g TCM‐199, 0.05 g NaHCO_3_, 0.75 g HEPES, 1.76 g NaCl, 3.00 g bovine serum albumin [BSA], 1 ml gentamicin, 1,000 ml Milli‐Q H_2_O) for searching. Metaphase‐II (MII) oocytes with extrusion of the first polar body were selected for IVF or SCNT.

### 4.4 In vitro fertilization and embryo culture

MII oocytes were washed and placed into 50 μl droplets of IVF medium (modified Tris‐buffered medium containing 2 mg/ml fatty acid‐free bovine serum albumin [FAF‐BSA] and 2 mM caffeine) in a mineral oil overlay and were maintained at 38.5°C until sperm were added. All IVF experiments used sperm obtained from a single domestic boar. A 0.1 ml frozen semen pellet was thawed in 3 ml of sperm‐washing medium (Dulbecco's phosphate‐buffered saline [DPBS] supplemented with 0.1% FAF‐BSA and 10‐μg/ml gentamicin). Sperms were washed by centrifugation in 45% Percoll solution and then in a modified Tris‐buffered medium. The spermatozoa pellet was resuspended in IVF medium to 0.5 × 10^6^ cells/ml. Then, 50 μl of the sperm suspension was added to the droplets to obtain a final concentration of 0.25 × 10^6^ cells/ml. Gametes were incubated together in a humidified incubator at 38.5°C for 4 hr (Spate et al., 2015).

Fertilized oocytes were removed, washed, and transferred in groups of 50 after IVF into two four‐well dishes containing 500 µl of two different variations of porcine zygote medium 3 containing 1.69mM arginine and 5μM PS48 (MU2; Redel, Tessanne, Spate, Murphy, & Prather, 2015; Spate et al., 2015): one with 5 mM +HT and one with 0 mM −HT. Embryos were further split into the culture in an atmosphere of 5% CO_2_ in air (high O_2_) or 5% O_2_, 5% CO_2_, and 90% N_2_ (low O_2_) at 38.5°C until Day 6 (D6) postfertilization. Thus, the four treatment groups were denoted as low O_2_ +HT (control), low O_2_ −HT, high O_2_ +HT, and high O_2_ −HT. The percentage cleaved on D2 and percentage developed to blastocyst stage on D6 were recorded for each treatment. Blastocyst‐stage embryos were fixed in 2% paraformaldehyde for 30 min at room temperature, stained with Hoechst 33342 (10 μg/ml) for 15 min, and the total number of nuclei was recorded after visualization by using an ultraviolet filter attached to a Nikon Eclipse E600 microscope (Nikon, Tokyo, Japan).

### 4.5 Embryo transfer and fetal collection

IVF‐derived D5 blastocysts and morulae cultured in low O_2_ −HT were placed in 3 ml of manipulation medium (9.50 g TCM‐199, 0.05 g NaHCO3, 0.75 g HEPES, 1.76 g NaCl, 3.00 g BSA, 1 ml gentamicin, 1,000 ml Milli‐Q H_2_O) with 5 μM PS48 in polystyrene tubes (BD Biosciences, San Jose, CA) and maintained at 37°C. At the University of Missouri Swine Research Complex, 60 embryos were loaded into a tomcat catheter and surgically transferred into the ampullary‐isthmic junction of a cycling gilt on D4 of her estrous cycle. Pregnancy was determined by heat checking and monitoring by ultrasound after D25. Due to space considerations in the facility, fetuses were collected on D42 of gestation, and crown–rump lengths and sexes based on external genitalia were recorded. To confirm the sexes of the fetuses, a porcine sex determination assay was performed by using genomic DNA extracted from the leg (Lucas et al., 2020). End‐point PCR was performed by using *DDX3* Forward 5′‐TGCTTGCTCGTGATTTCTTGGA and *DDX3* Reverse 5′‐GCCACTAGAATTGGGCTTTTTCCT with the conditions of 94°C for 30 s, 35 cycles of 94°C for 15 s, 64°C for 30 s, 72°C for 1 min, and a final extension of 72°C for 2 min. Porcine *DDX3* was amplified from both sex chromosomes, and amplicon lengths were 485 bp for the X chromosome and 545 bp for the Y chromosome.

### 4.6 RNA extraction and complementary DNA (cDNA) synthesis

D6 blastocyst‐stage embryos were washed with diethylpyrocarbonate (DEPC)‐treated phosphate‐buffered saline (PBS), collected in pools of 30 in 0.6‐ml tubes, and snap‐frozen in liquid nitrogen for storage at −80°C. Total RNA was extracted from each pool by using an RNeasy Micro kit (Qiagen, Germantown, MD), according to the manufacturer's instructions and eluted in 12 μl of nuclease‐free water. The SuperScript VILO kit (Life Technologies, Carlsbad, CA) was used to convert to cDNA according to the manufacturer's instructions.

### 4.7 Quantitative PCR

qPCR was performed with each cDNA sample as a template for transcripts involved in HT synthesis, oxidative stress, and apoptosis by using the IQ SYBR Green Supermix (Bio‐Rad Laboratories, Hercules, CA). Primers were designed by using Integrated DNA Technology software (Idtdna.com; Coralville, IA) and are listed in Table 4. Efficiency tests were conducted for each primer set by generating a standard curve of 10 ng dilutions from a 50 ng/μl pooled cDNA reference sample. qPCR was conducted in triplicate on the CFX Connect™ Real‐Time System (Bio‐Rad Laboratories) for each concentration (50, 5, 0.5, 0.05, and 0.005 ng/μl) for validation of each set of primers. Accepted primer sets had standard curve *R* ^2^ values of ≥0.99 with efficiencies of 95–105%. Samples from every biological replicate were diluted to 5 ng/μl, and quantitative PCR was run in triplicate to determine the expression of the selected transcripts with the conditions: 95°C for 3 min, and 40 cycles of 95°C for 10 s, 55°C for 10 s, and 72°C for 30 s. A dissociation curve was generated after amplification to ensure that a single product was amplified.

**Table 4:** Transcripts selected for quantitative polymerase chain reaction

Gene	Forward primer	Reverse primer	Accession number
*CSAD*	5′‐CCTGCAACCTCATGGTTATT	5′‐AGAGTGAGTGGGTCAAAGTA	XM_021091700.1
*SOD1*	5′‐AGGGAGAGAAGACAGTGTTAG	5′‐CTGGTACAGCCTTGTGTATTAT	NM_001190422.1
*GPX6*	5′‐AAACAAGAACCAGCAAAGAAC	5′‐GGAGTTCTTCAGGAAGGTAAAG	NM_001137607.1
*BAD*	5′‐TTGCCAGCCGAGATTAACCCTAAC	5′‐CACGCGGGCTTTATTAGCACGTTT	XM_021082883.1
*CASP3*	5′‐CTACAGCACCTGGTTACTATTC	5′‐TTATGC ACATTCTTACTCGGG	NM_214131.1
*YWHAG*	5′‐TCCATCACTGAGGAAAACTGCTAA	5′‐TTTTTCCAACTCCGTGTTTCTCTA	XM_005661962.3

The abundance of each mRNA transcript was calculated relative to the housekeeping gene, *YWHAG* (tyrosine 3‐monooxygenase/tryptophan 5‐monooxygenase activation protein, gamma polypeptide) and a reference sample, which consists of pooled cDNA from various porcine tissues (Whitworth et al., 2005). The comparative threshold cycle method (*C*_q_) method was used to determine transcript abundance for each treatment.

### 4.8 FLICA assay

D6 blastocyst‐stage embryos were incubated in a 3× FLICA probe for 2 hr to stain activated caspases 3 and 7 (MilliporeSigma, Burlington, MA). Afterward, the embryos were fixed in 2% paraformaldehyde for 30 min, and nuclei were stained with Hoechst 33342 (10 μg/ml) for 20 min at room temperature. Negative control groups were incubated in the concentration of dimethyl sulfoxide (DMSO) used to resuspend the FLICA probe to 150× and diluted to 3× in TL‐HEPES. Positive control groups were incubated in a 3× FLICA probe with 10 μM of staurosporine. Blastocysts were mounted on glass slides and imaged by using epifluorescence illumination. Caspase activity of each blastocyst was calculated as the percentage of FLICA‐positive cells out of the total number of cells.

### 4.9 TUNEL assay

D6 blastocyst‐stage embryos from each treatment group were fixed in 2% paraformaldehyde for 30 min and permeabilized with 0.5% Triton X‐100 for 1 hr at room temperature. Apoptotic nuclei in the blastocysts were labeled by incubating in 25 μl of TUNEL solution (fluorescein‐conjugated dUTP and terminal deoxynucleotidyl transferase) for 1 hr at 38°C (In Situ Cell Death Detection Kit; Roche Diagnostics, Mannheim, Germany). Negative control groups were incubated with only the “label” solution containing fluorescein‐conjugated dUTP. Positive control groups were incubated with 16 Kunitz units of DNase I in 25 μl of TUNEL solution. Blastocysts were washed three times in TL‐HEPES, and all nuclei were stained with Hoechst 33342 (10 μg/ml) for 20 min at room temperature. Then, the blastocysts were mounted on glass slides and imaged by using epifluorescence illumination. The percentage of DNA fragmentation in each blastocyst was calculated as the percentage of TUNEL‐positive nuclei out of the total number of nuclei.

### 4.10 Fetal fibroblast cell culture and somatic cell nuclear transfer

Porcine fetal fibroblasts were collected from Day 35 wild‐type fetuses and were stored in cryovials at 1.5 × 10^6^ cells/ml in 0.5 ml of 90% fetal bovine serum (FBS) and 10% DMSO. Cells were thawed and cultured in Dulbecco's modified Eagle's medium (1 g/L glucose, glutamine, and pyruvate with phenol red) supplemented with 15% FBS and 10 µg/ml of gentamicin for 4 days in T25 flasks (Corning, Corning, NY) at 38.5°C in a humidified incubator with an atmosphere of 5% O_2_, 5% CO_2_, and 90% N_2_. Cells were rinsed with DPBS and dissociated from flasks by 4 min incubation (37°C) with 0.05% Trypsin–ethylenediaminetetraacetic acid (Gibco, Denmark). Fibroblasts were centrifuged for 5 min at 500*g* and resuspended in manipulation medium with 7.0 µg/ml of cytochalasin B for SCNT.

Procedures for SCNT have been previously described (Lai & Prather, 2003). Briefly, MII oocytes were placed in 7 μl drops of manipulation medium with 7.0 μg/ml of cytochalasin B on a micromanipulator with an inverted microscope and enucleated by aspirating the polar body, MII plate, and its surrounding cytoplasm by using a hand‐tooled beveled glass pipette. A single donor cell was injected into the perivitelline space next to the oocyte membrane. Then, the oocyte and donor cell were placed in fusion medium (0.3 M mannitol, 0.1 mM CaCl_2_, 0.1 mM MgCl_2_, 0.5 mM HEPES buffer, pH 7.2) and fused by two direct current pulses (1‐s interval) at 1.2 kV/cm for 30 μs by using a BTX Electro Cell Manipulator (Harvard Apparatus, Holliston, MA). Reconstructed embryos were activated with 200‐μM thimerosal for 10 min in the dark and 8‐mM dithiothreitol for 30 min. Afterward, the embryos were split into two groups and placed in MU2 +HT or −HT with 0.5 μM of scriptaid, a histone deacetylase inhibitor, for 14–16 hr at 38.5°C and 5% CO_2_ in air (Whitworth, Zhao, Spate, Li, & Prather, 2011). Embryos were placed into the respective media without scriptaid and cultured to D6 in 5% O_2_, 5% CO_2_, and 90% N_2_. The percentage cleaved on D2, percentage developed to blastocyst stage on D6, and the total number of nuclei were recorded for each treatment.

### 4.11 Statistical analysis

All experiments were repeated at least four times so that the replicate variation could be assessed. For IVF experiments, percentage data used to quantify embryo cleavage and development to the blastocyst stage was analyzed by a generalized linear model (PROC GENMOD). The total number of nuclei, transcript abundance ($2^{-\Delta \Delta C_{t}}$), caspase activity, and DNA fragmentation were analyzed by linear mixed models (PROC MIXED). Analyses for developmental parameters and DNA fragmentation of SCNT embryos were conducted by using Student's *t* test. The Shapiro–Wilk test was used for assessing the normality assumption for each experiment and none of the data deviated from the normality assumption. Treatment was modeled as a fixed factor and the biological replicate was modeled as a random factor. Significance was discovered by testing hypotheses by using least square estimates. The type I error and family‐wise error rate were controlled at a level of 0.05. All of these analyses were conducted by using SAS version 9.4 (SAS Institute, Cary, NC).

## CONFLICT OF INTERESTS

The authors declare that there are no conflict of interests.

## AUTHOR CONTRIBUTIONS

P. R. C., L. D. S., and R. S. P. conceived and designed the experiments for the study. P. R. C., L. D. S., E. C. L., J. A. B., R. C. F., and T. K. H. conducted the experiments. P. R. C. analyzed the data and wrote the manuscript. All authors revised and accepted the manuscript.
